# The Correlation between High-Sensitivity C-Reactive Protein (hsCRP) Serum Levels and Severity of Coronary Atherosclerosis

**Published:** 2014-01-01

**Authors:** Mohammad Assadpour Piranfar

**Affiliations:** 1 Department of Cardiology, Taleghani Hospital, Shahid Beheshti University of Medical Sciences, Tehran, IR Iran

**Keywords:** Coronary Stenosis, Atherosclerosis, Cardiac Imaging

## Abstract

**Background:**

Considering the increasing incidence of coronary artery stenosis as well as its related complications, the importance of its etiology, and inconsistent reports, we aimed to determine the relationship between High-sensitivity C-reactive protein (hsCRP) serum levels and severity of coronary atherosclerosis.

**Methods:**

This cross-sectional study was conducted on the patients who referred to Taleghani Hospital, Tehran, Iran and met the inclusion criteria in 2011. Regarding the severity of the disease, the angiographic findings were categorized to mild (< 10), moderate (10 - 50), and severe (> 50) using the Gensini score classification. 1 mL blood sample was taken from each patient and transferred to the laboratory after clotting. After centrifugation, the serum hsCRP level was measured and classified in 3 levels of 1, 1 to 3, and more than 3 mg / L. The relationship between hsCRP serum levels and the severity of coronary atherosclerosis was analyzed using Chi-square test (N = 85, P value < 0.010).

**Results:**

This study was performed on 85 patients with the mean age of 55.7 ± 7.06 years. Besides, 64.7% of the participants were male. According to the results, 34.1%, 37.7%, and 28.2% of the patients experienced mild, moderate, and severe disease intensity, respectively. Moreover, the serum hsCRP levels were < 1, between1 and 3, and > 3 mg / L in 28.2%, 27.1%, and 44.7% of the patients, respectively. The hsCRP serum levels were significantly higher in the patients with moderate and severe artery stenosis compared to those with mild stenosis (P < 0.010).

**Conclusions:**

The hsCRP serum levels were significantly related to the severity of coronary atherosclerosis.

## 1. Background

The severity of coronary atherosclerosis is one of the main causes of referral to clinics and hospitals. It has been reported that 90% of the patients who referred to the emergency department complained about chest pain. Chest pain is the cause of about 6 million referrals to Emergency Rooms (ERs) in the United States each year. In general, factors such as lipid profile, diabetes mellitus, smoking, age, gender, and genetics have been introduced as risk factors for coronary atherosclerosis (1). However, if all the risk factors and markers are not recognized, symptomatic treatment may not be effective probably leading to mortality (2, 3).

Previous studies on the relationship between High sensitivity C-reactive protein (hsCRP) and severity of coronary atherosclerosis have revealed controversial results (4-9).

Considering these controversies, the increasing incidence and severity of coronary atherosclerosis, and importance of knowing about other markers, the present study aims to determine the correlation between serum hsCRP levels and severity of coronary atherosclerosis in a sample of Iranian patients referring to Taleghani Hospital in 2011.

## 2. Materials and Methods

This cross-sectional study was performed on 85 patients who had referred to the cardiac clinic in Taleghani Hospital, Tehran, Iran due to chest pain in 2011 with positive Thalium heart scan for ischemia. All the selected cases met the inclusion criteria of the study. The patients with creatinine > 1.5 mg / dL, recent acute coronary syndrome, acute or chronic liver disease, infectious or non-coronary inflammatory disease, and symptomatic heart failure were excluded from the study. The study participants underwent angiography using standard techniques (10) and the severity of coronary atherosclerosis was categorized into 3 levels of mild (< 10), moderate (10 - 50), and severe (> 50) based on the Gensini score (11).

The patients' demographic variables, such as age and sex, and angiography results were recorded. After explaining the aims of the study and obtaining the patients' approval for participation, 1 mL blood samples were taken from the patients and were transferred to the laboratory after clotting. After centrifugation (DBC kit, Canada), the serum hsCRP level was measured and classified as < 1, 1 - 3, and ≥ 3 mg / L. The relationship between the hsCRP levels and the severity of atherosclerosis was analyzed using Chi-square test. Ultimately, the collected data were analyzed using the SPSS statistical software, version 16. Besides, P value < 0.05 was considered as statistically significant.

## 3. Results

This study was conducted on 85 candidates for coronary angiography with the mean age of 55.7 ± 7.06 years. Among the participants, 55 (64.7%) were male and 30 (35.3%) were female. The serum hsCRP levels were < 1, 1 - 3, and ≥ 3 mg / L in 24 (28.2%), 23 (27.1%), and 38 (46.7%) patients, respectively.

According to the angiography findings, the severity of atherosclerosis was mild in 29 (34.1%), moderate in 32 (37.7%), and severe in 24 patients (28.2%).

Table 1 shows the distribution of the participants in terms of severity of coronary artery involvement based on serum hsCRP levels. Accordingly, 1 patient (1.2%) with serum hsCRP level < 1 mg / L had severe coronary atherosclerosis. Also, 7 patients (8%) with 1 - 3 mg / L serum hsCRP levels and 16 ones (18.4%) with serum hsCRP levels ≥ 3 mg / L had severe coronary atherosclerosis (P < 0.010).

**Table 1.** Frequency (%) of the Patients in Terms of Severity of Their Disease and Serum hsCPR Levels

hsCRP	Mild (< 10)	Moderate	Severe (> 50)	Total	P value
	**(n = 29)**	**(n = 32**)	**(n = 24)**	**(n = 85)**	
**< 1 mg / L**	15 (16.7)	8 (9.4)	1 (1.2)	24 (28.2)	< 0.010
**1 - 3 mg / L**	14 (16.5)	2 (2.4)	7 (8.2)	23 (27.1)	
**≥ 3 mg / L**	0	22 (25.9)	16 (18.8)	38 (44.7)	< 0.010

## 4. Discussion

The results of this study showed a significant relationship between the severity of coronary stenosis and serum hs-CRP level. In a recent similar study on 80 patients in India also, a statistically significant correlation was observed between the increased serum hsCRP levels and Gensini score (6). The publication of that article as the present study was being performed indicates the novelty of the subject.

Moreover, Luo J G and colleagues investigated the relationship between serum hsCRP and TNFα levels and severity of coronary atherosclerosis in 100 patients. They found a positive and effective correlation between the mentioned indices and the Gensini score (12).

In another study by Mahjan on 140 patients with premature coronary artery disease in India, the researchers found a positive correlation between the serum levels of inflammatory markers and the severity of coronary atherosclerosis in the patients without diabetes mellitus. Nevertheless, since only non-diabetic patients who had premature coronary atherosclerosis were studied, the findings could not be generalized (13).

In contrast, the study Ulucay conducted on 51 patients revealed no significant relationship between the serum hsCRP levels and the severity of coronary atherosclerosis (8). This finding may be attributed to the small sample size of the study. The discussions of reliability and measurement could have impacted the findings, as well. One other possibility is that the angiorgraphy films might have been read by students or unskilled people.

Generally, hsCRP is correlated with cardiovascular diseases through two inflammatory processes:

1) Production of hsCRP by atheromatous tissue or vascular smooth muscles of coronary arteries.

2) Production of hsCRP stimulated by lipid tissues cytokines.

On the other hand, the produced hsCRP is able to activate the circulating leukocytes. As a result, atheroma plaque stabilizing mediators, such as Matrix Metalloproteinase (MMP-9), Monocyte Chemotactic Protein 2 (MCP2), and urokinase-type Plasminogen Activator (uPA) are produced and released.

HsCRP is able to reduce nitric oxide activity in endothelial cells eventually leading to disorders in the vasodilation of the arteries. However, its relationship with the severity of coronary atherosclerosis is still questionable. The current study provided us with the evidence that the severity of the disease could be predicted based on the hsCRP values. Therefore, this index is better to be taken into account.

Our study had some limitations. We could have come to more precise findings if we had performed an analytical study with a control group instead of a cross-sectional study. Thus, we suggest further studies to include control groups, as well. Besides, although we used similar cut-off points, it is not clear whether these cut-off values are the same in different countries and evaluations should have been done in this regard. Cut-off values may change with the society's particular health and economic conditions. Moreover, since the Gensini score was calculated based on reading the angiography films, probable observer errors might have occurred. Therefore, to avoid such errors, the angiography films should be read by experts. Furthermore, since the participants were enrolled in an educational hospital, most patients were from middle to low social classes and this is another factor which limits the findings of the study.

Yet, to the best of our knowledge, no such study has been done in Iran up to now. The novelty of our study, its controversy, and its strength concerning the effect of regional factors with the regard to life conditions make the study distinguished.

The findings of the present study indicated a significant correlation between serum hsCRP and the severity of coronary artery involvement. Hence, further analytical researches are recommended to be conducted on this issue.
